# The clinical meaning of lymphovascular invasion: preoperative predictors and postoperative implications in prostate cancer - a retrospective study

**DOI:** 10.3389/fonc.2024.1349536

**Published:** 2024-05-03

**Authors:** Jakub Karwacki, Małgorzata Łątkowska, Michał Jarocki, Arkadiusz Jaworski, Przemysław Szuba, Adrian Poterek, Artur Lemiński, Krystian Kaczmarek, Agnieszka Hałoń, Tomasz Szydełko, Bartosz Małkiewicz

**Affiliations:** ^1^Department of Minimally Invasive and Robotic Urology, University Center of Excellence in Urology, Wrocław Medical University, Wrocław, Poland; ^2^Department of Ophthalmology, Wrocław Medical University, Wrocław, Poland; ^3^Faculty of Economics in Opole, WSB University in Wroclaw, Wroclaw, Poland; ^4^Department of General and Oncologic Urology, Independent Public Regional Hospital in Szczecin, Szczecin, Poland; ^5^Department of Biochemical Sciences, Pomorenian Medical University, Szczecin, Poland; ^6^Department of Clinical and Experimental Pathology, Wroclaw Medical University, Wroclaw, Poland; ^7^University Center of Excellence in Urology, Wrocław Medical University, Wrocław, Poland

**Keywords:** prostate cancer, radical prostatectomy, lymphovascular invasion, histopathological examination, oncologic staging, prognostic factors

## Abstract

**Introduction:**

Lymphovascular invasion (LVI) is a pivotal histopathological parameter in prostate cancer (PCa), holding significant prognostic implications. Our study pursued a dual objective: firstly, to identify preoperative factors associated with LVI, aiming to unveil markers facilitating the recognition of patients prone to LVI during postoperative examination; and secondly, to assess postoperative outcomes correlated with LVI.

**Methods:**

We retrospectively analyzed 861 nonmetastatic PCa patients who underwent radical prostatectomy (RP), investigating preoperative factors and postoperative outcomes. Surgical specimens were processed following established guidelines. Statistical analyses utilized non-parametric tests to assess the association between LVI and both pre- and postoperative factors. Furthermore, logistic regression analyses were utilized to develop models aimed at identifying the most significant predictors of LVI and pN1 status, respectively.

**Results:**

Numerous preoperative factors exhibited significant correlations with LVI, offering valuable clinical insights. Logistic regression identified magnetic resonance imaging (MRI)-based clinical tumor stage (cT) 3-4, biopsy Gleason Grading Group (GGG) 3-5, preoperative prostate specific antigen (PSA) ≥20 and percentage of positive biopsy cores (PPBC) ≥50% as the strongest preoperative predictors of LVI. Additionally, the study uncovered an association between LVI and postoperative outcomes, including postoperative PSA (*p* value <0.001), extracapsular extension (ECE) (<0.001), positive surgical margins (PSM) (<0.001), perineural invasion (PNI) (<0.001), pathological tumor stage (pT) (<0.001), pathological lymph node status (pN) (<0.001), postoperative GGG (<0.001), and operative time (0.023). Notably, the study revealed a novel and substantial association between LVI and an increased number of positive lymph nodes in pN+ patients in the univariate analysis (<0.001). Furthermore, we have found an association between LVI and pN1 status in the logistic regression analysis (odds ratio [OR] = 23.905; *p <*0.001).

**Conclusion:**

Our findings underscore the pivotal role of LVI in influencing the prognosis of prostate cancer (PCa). The study acknowledges the challenges associated with preoperative LVI assessment and emphasizes the need for future research to unravel the factors associated with this histopathological finding. Significantly, our research stands out as the first, to the best of our knowledge, to reveal the association between LVI and the number of positive lymph nodes in pN+ patients.

## 1 Introduction

Lymphovascular invasion (LVI), also known as microvascular invasion (1–3) or vessel tumor embolus (4–6), is most often defined as the unequivocal presence of tumor cells within endothelial-lined spaces (7). In the context of prostate cancer (PCa), the second most common malignancy in men worldwide (8), LVI has emerged as a pivotal histopathological parameter with significant prognostic implications.

The pathological evaluation of radical prostatectomy (RP) specimens assumes paramount importance in predicting patient outcomes accurately. Traditionally, key histopathological determinants such as Gleason score, pathological tumor (pT) stage, lymph node status (pN), or surgical margin status have informed prognostication. However, multiple systematic reviews and meta-analyses consistently demonstrate that the presence of LVI in final histopathology (pL1) is associated with adverse clinical outcomes, including higher rates of biochemical recurrence, diminished survival rates, and an increased likelihood of unfavorable histopathological features such as perineural invasion (PNI), positive surgical margins (PSM), and nodal involvement (7, 9–11). In cases where evidence of LVI is identified within a prostate cancer needle biopsy specimen, it is recommended that patients forego active surveillance (AS) and opt for radical treatment (12, 13). The presence of LVI in the final histopathological assessment is considered unfavorable, as it is associated with other adverse pathological outcomes and unfavorable survival rates, as indicated by the guidelines of the European Association of Urology (EAU) (13).

The controversy surrounding LVI primarily emanates from the reliance on retrospective studies for data analysis. Furthermore, it is exacerbated by instances where LVI exhibits significance in predicting biochemical recurrence (BCR) only within univariate settings but loses significance in multivariate analyses, as observed in certain studies (4, 14, 15). Additionally, the issue is compounded by the considerable variability in reported LVI frequencies, ranging widely from 3.6% to 53% (16, 17).

Our study aims to address the existing gaps in the literature by focusing on the pivotal role of LVI in PCa prognosis. Specifically, our primary objective is to identify preoperative factors associated with the presence of LVI, shedding light on potential predictive markers in the context of PCa. Additionally, our research group endeavors to elucidate the intricate relationship between LVI and adverse histopathological outcomes, thereby enhancing our understanding of the broader implications of LVI in this context. Through our investigation, we aim to contribute valuable insights to the field, ultimately advancing the clinical management and prognostication of PCa patients.

## 2 Materials and methods

### 2.1 Patient population and clinicopathological data

We analyzed 861 patients with histologically confirmed nonmetastatic PCa who underwent RP at University Center of Excellence in Urology in Wrocław, Poland, between September 2012 and November 2021. The exclusion criteria comprised missing LVI status (pLx). We decided not to exclude patients with the history of neoadjuvant therapy. In pursuit of maximizing our dataset, we opted to include patients with missing data. The patient counts (n) with available data for each covariate can be found in **Tables 1**, **2**, situated adjacent to the respective variables.

**Table 1:** Association of lymphovascular invasion with preoperative clinicopathological parameters in 861 patients who underwent radical prostatectomy.

Characteristic	*n*	pL0 (*n* = 709; 82.3%)	*n*	pL1 (*n* = 152; 17.7%)	*p* value
Demographic characteristics					
Age at RP, years	708	64.1 ± 6.3, 65 (31-80)	152	63.9 ± 6.6, 64 (41-78)	0.714
Body mass, kg	602	85.4 ± 13.4, 85 (52-130)	136	87.3 ± 13.5, 85 (52-140)	0.230
BMI	595	27.9 ± 3.9, 27.7 (18-43.9)	134	28.3 ± 4, 27.9 (18.4-45.2)	0.414
Pack-years (in smokers)	125	21.4 ± 13.5, 20 (2-80)	27	25 ± 16, 20 (0.6-60)	0.275
Clinical parameters					
Biopsy PSA, ng/ml	458	11.6 ± 10.6, 8.4 (1-104)	87	22.3 ± 22.8, 17 (2.8-149)	**<0.001**
Preoperative PSA, ng/ml	664	11.6 ± 10.4, 8.6 (0-97.5)	145	24.6 ± 23.3, 18.7 (0.4-174)	**<0.001**
Testosterone, ng/ml	338	3.7 ± 1.6, 3.5 (0.1-9.6)	63	3.9 ± 1.9, 3.8 (0.1-9)	0.400
Albumins, ng/ml	313	4.4 ± 0.3, 4.5 (3.5-5.7)	52	4.4 ± 0.3, 4.4 (3.3-4.9)	0.064
Preoperative Hgb, g/dl	692	14.1 ± 1.3, 14.9 (9.4-19)	147	14.7 ± 1.2, 14.9 (10.2-17.3)	0.493
PLR	304	139.8 ± 55.5, 129.9 (7.5-335.6)	52	139.6 ± 52, 133.8 (61.8-274.7)	0.933
NLR	307	3.2 ± 3.4, 2.6 (0.4-38.2)	52	3.5 ± 4.6, 2.5 (0.9-34.6)	0.984
Clinical T stage					**<0.001**
cT1	77	96.3%	3	3.7%	
cT2	523	85.9%	86	14.1%	
cT3	66	54.5%	55	45.5%	
cT4	1	25%	3	75%	
Pathological parameters					
Biopsy GGG					**<0.001**
GGG1	372	92.8%	29	7.2%	
GGG2	153	84.1%	29	15.9%	
GGG3	67	71.3%	27	28.7%	
GGG4	74	72.5%	28	27.5%	
GGG5	28	46.7%	32	53.3%	
PPBC, %	364	35 ± 23, 31 (0-100)	86	51 ± 29, 58 (0-100)	**<0.001**
Imaging parameters					
Preoperative scintigraphy					0.823
no changes	301	82.9%	62	17.1%	
3 or less changes	11	68.8%	5	31.3%	
over 3 changes	2	100%	0	0%	
MRI T stage					**<0.001**
cT1	16	100%	0	0%	
cT2	137	85.1%	24	14.9%	
cT3	20	52.6%	18	47.4%	
cT4	1	100%	0	0%	
MRI N stage					**0.003**
cN0	177	82.3%	38	17.7%	
cN1	5	45.5%	6	54.5%	
Prostate volume, ml	544	40.5 ± 19.6, 36 (2-220)	107	42 ± 18.8, 40 (15-130)	0.239
Other					
EAU risk group					**<0.001**
1	146	96.1%	6	3.9%	
2	179	94.2%	11	5.8%	
3	234	75.5%	76	24.5%	
4	54	55.1%	44	44.9%	

All continuous data is presented as mean ± SD and/or median (range). All interval data is presented as number and percent. n, number of patients; pL0, patients without lymphovascular invasion in final histopathology; pL1, patients with lymphovascular invasion in final histopathology; RP, radical prostatectomy; BMI, body mass index; PSA, prostate specific antigen; Hgb, hemoglobin; PLR, platelet-to-lymphocyte ratio; NLR, neutrophil-to-lymphocyte ratio; cT, clinical tumor stage; GGG, Gleason Grading Group; PPBC, percentage of positive biopsy cores; MRI, magnetic resonance imaging; cN, clinical lymph node status; EAU, The European Association of Urology.

**Table 2:** Association of lymphovascular invasion with various peri- and postoperative clinicopathological parameters in 861 patients who underwent radical prostatectomy.

Characteristic	*n*	pL0 (*n* = 709; 82.3%)	*n*	pL1 (*n* = 152; 17.7%)	*p* value
Clinical parameters					
Blood loss, ml	656	657.7 ± 413.1, 600 (0-3,300)	143	733 ± 520.6, 600 (100-3,600)	0.244
Blood transfusion	695	0.2 ± 0.8, 0 (0-10)	151	0.3 ± 1.1, 0 (0-8)	0.278
Postoperative Hgb, g/dl	673	12.3 ± 1.3, 12.3 (6.7-16.6)	142	12.2 ± 1.4, 12.2 (7.3-16.8)	0.254
Postoperative PSA, ng/ml	342	0.2 ± 1.3, 0 (0-18)	95	1.4 ± 4.5, 0.1 (0-35)	**<0.001**
Histopathological parameters					
ECE					**<0.001**
negative	475	95.4%	23	4.6%	
focal	115	74.7%	39	25.3%	
diffuse	115	56.4%	89	43.6%	
Surgical margin					**<0.001**
negative	376	90.8%	38	9.2%	
≤ 3 mm	139	79.4%	36	20.6%	
> 3 mm	171	69.5%	76	30.5%	
PNI					**<0.001**
negative	75	97.4%	2	2.6%	
positive	608	81.1%	142	18.9%	
Pathological T stage					**<0.001**
pT1	4	100%	0	0%	
pT2	474	96.9%	15	3.1%	
pT3	226	62.4%	136	37.6%	
pT4	0	0%	1	100%	
Pathological N stage					**<0.001**
pN-	463	91.9%	41	8.1%	
pN+	36	25.2%	107	74.8%	
Postoperative GGG					**<0.001**
GGG1	121	99.2%	1	0.8%	
GGG2	276	92.6%	22	7.4%	
GGG3	164	78.8%	44	21.2%	
GGG4	62	79.5%	16	20.5%	
GGG5	76	53.5%	66	46.5%	
Positive LNs in pN+ patients	36	1.9 ± 2.1, 1 (1-12)	107	4.07 ± 4.8, 3 (1-31)	**<0.001**
Other					
Operative time, minutes	665	174.3 ± 47.8, 165 (60-375)	148	184.6 ± 49.4, 180 (75-375)	**0.023**

All continuous data is presented as mean ± SD and/or median (range). All interval data is presented as number and percent. n, number of patients; pL0, patients without lymphovascular invasion in final histopathology; pL1, patients with lymphovascular invasion in final histopathology; Hgb, hemoglobin; PSA, prostate specific antigen; ECE, extracapsular extension; PNI, perineural invasion; pT, pathological tumor stage; pN, pathological lymph node status; GGG, Gleason Grading Group; LNs, lymph nodes.

The clinical T stage was assessed according to the TNM classification from 2016 (18); the prostate biopsy was obtained by TRUS-guided systematic, targeted, or combined biopsy. The following baseline characteristics and clinical parameters were retrospectively collected and evaluated for each patient. Preoperative data included: age at the time of surgery, body weight and body mass index (BMI), smoking status and pack-years, biopsy serum PSA (PSA at diagnosis) and preoperative serum PSA level, testosterone level, albumin level, preoperative hemoglobin level, platelet-to-lymphocyte ratio (PLR), neutrophil-to-lymphocyte ratio (NLR), biopsy Gleason score (Gleason Grading Groups, GGG), percentage of positive systematic biopsy cores (PPBC), clinical T (cT) stage assessed after digital rectal examination (DRE), scintigraphy results, cT and cN stages proposed after MRI, prostate volume, and EAU risk group classification.

The surgical approach for RP involved either open with an ascending technique or laparoscopic with extra- or transperitoneal access. In both cases, a modified-extended pelvic lymphadenectomy (mePLND) was performed (19), encompassing the removal of tissues around the obturator fossa, internal and external iliac arteries, extending to the distal part of the common iliac artery, as well as presacral regions and Marcille’s fossa. Peri- and postoperative data we collected included: blood loss, blood transfusion, postoperative hemoglobin, postoperative PSA, extracapsular extension (ECE), surgical margins, PNI, LVI status (pL0 or pL1), pathological T (pT) and pN stages, postoperative Gleason scores, number of positive lymph nodes in pN+ patients, and operative time.

### 2.2 Pathologic examination

Surgical specimens were collected and processed according to the Stanford protocol guidelines. The specimens were fixed in a neutral buffered formalin solution and embedded in paraffin. Tissue samples were sectioned using a microtome and stained with hematoxylin and eosin (H&E). Experienced uropathologists evaluated the sample slides and documented the results based on a standardized reporting system. Pathological stages were defined according to the American Committee’s guidelines for the Staging System for Prostate Cancer (20), and Gleason scores were determined following the International Society of Urological Pathology (ISUP) PCa grading consensus (21). Detailed pathological findings for the presence of LVI, PNI, ECE, and surgical margins were also examined and documented. LVI was defined as the unequivocal presence of tumor cells within endothelial-lined spaces with no underlying muscular walls (22) or the presence of tumor emboli in small intraprostatic vessels (23). The analysis of LVI included evaluations of both prostate and seminal vesicle specimens. In our study cohort, all patients showed LVI exclusively in prostate specimens, with no instances observed in seminal vesicles. While the presence of LVI in seminal vesicles was not a specific exclusion criterion, it is a rare event based on our center’s experience. In cases where diagnostic uncertainty arose, podoplanin (D2-40 or PDPN) staining was utilized to aid uropathologists in their decision-making process.

### 2.3 Statistical analysis

All statistical analyses were carried out using PS Imago Pro 9.0, 2022, polish license. Data were expressed as means ± SD and/or median (range) for continuous variables and number (percentage) for categorical variables. To assess the normal distribution of variables, we employed the Shapiro-Wilk tests (24). The distribution of all variables subjected to analysis significantly deviated from a normal distribution. Consequently, the research team relied on nonparametric measures with lower formal requirements. Specifically, the U-Mann-Whitney test was used to compare mean levels between two groups with dichotomous variables. The Kruskal-Wallis test assessed differences in mean levels among groups with categorical variables, each having at least three levels. Additionally, Kendall’s tau-b coefficient was applied to determine if two variables can be considered statistically dependent. In all cases, a two-sided testing approach was applied. Statistically significant differences between groups occur when the test statistic (p-value) is less than 0.05. Additionally, logistic regression made it possible to identify significant preoperative factors that are the strongest predictors of LVI. In the analyses, a stepwise estimation method was employed, involving the iterative selection of variables for the model. In the initial step, the model is computed for all potential variables. Subsequent iterations eliminate the least fitting variables that disrupt the model’s significance and coefficient of determination. Ultimately, the method enables the attainment of the most optimal model tailored to the selected variables. Initially, six models were tested, each comprising seven variables (cT, cN, biopsy GGG, biopsy PSA (or PSA at diagnosis), preoperative PSA, PPBC, and MRI based cT), which were appropriately coded. Furthermore, we conducted an additional logistic regression analysis to identify significant factors that serve as predictors of pN1 status, aiming to evaluate the significance of LVI as a potential determinant for nodal involvement. This model incorporated eight variables, including postoperative GGG, pT stage, PPBC, preoperative PSA level, ECE, PSM, PNI, and LVI. Additionally, we carried out a linear regression analysis to identify predictors associated with the number of positive lymph nodes in pN1 patients.

## 3 Results

Mean patient age at diagnosis was 64.1 years (range 31 to 80), and mean PSA was 14.0 ng/mL. The mean percent of positive biopsy cores (PPBC) was 39.7% (range 0 to 100%). 4 (0.5%) of the 861 patients had pT1 disease after histopathological examination, 489 (57.1%) had pT2 disease, 362 (42.3%) had pT3 disease, and 1 patient (0.1%) had pT4 disease. Of 647 patients, 143 (22.1%) showed lymph node involvement. In the whole analyzed cohort of 861 patients, 152 (17.7%) obtained pL1 status in the final histopathology.

**Tables 1**, **2** show the association of LVI with pre- and postoperative clinicopathological variables. On univariate analyses, LVI was associated with higher PSA at diagnosis (biopsy PSA; *p* < 0.001), preoperative PSA (<0.001), clinical T stage (<0.001), biopsy GGG (<0.001), PPBC (<0.001), clinical T stage assessed with MRI (<0.001), MRI N stage (0.003), the EAU risk group (<0.001), postoperative PSA (<0.001), ECE (<0.001), PSM (<0.001), PNI (<0.001), higher pT stage (<0.001), pN stage (<0.001), postoperative GGG (<0.001), higher number of positive LNs in pN+ patients (<0.001), and with higher operative time (0.023).

The results of the logistic regression, conducted with the backward elimination method, are presented in **Table 3**. Among the identified predictors, MRI findings denoting clinical stage cT3-4, biopsy GGG of 3-5, preoperative PSA levels ≥20, and PPBC exceeding 50% emerged as the most influential factors. In the multivariate analysis, it is notable that both biopsy GGG and preoperative PSA levels were not statistically significant, as indicated by their respective p-values of 0.051 and 0.077. Nevertheless, patients with GGG 3-5 exhibited an odds ratio (OR) of 3.005 for having LVI, while patients with preoperative PSA levels exceeding 20 ng/ml demonstrated OR of 2.899. The logistic regression model underscored the significance of MRI clinical stage cT3-4 and a PPBC exceeding 50% as the strongest predictors. These factors exhibited OR of 4.739 and 7.364, respectively. The initial model incorporated three additional variables: clinical stage cT1-2 vs. cT3-4, cN0 vs. cN+, and biopsy PSA <20 vs. ≥20. Summary results for the regression model from **Table 3** were as follows: *n* = 861, percentage of correct classifications = 88.1%, Nagelkerke’s R2 = 0.458, Cox and Snell’s R2 = 0.287, Hosmer-Lemeshow Test = 0.208. To ensure robust and reliable results, a total of six different models were tested with varying coding schemes for the same variables, exploring different threshold levels. All six models are presented in the **Supplementary Table 1** in the **Supplementary Material**.

**Table 3:** Logistic regression results with LVI occurrence in a final histopathological examination as a dependent variable.

Predictors	B	Wald	Statistical significance	Exp(B)
Biopsy GGG 3-5	1.100	3.807	0.051	3.005
MRI cT3-4	1.556	7.128	0.008	4.739
PPBC >50%	1.997	10.058	0.002	7.364
Preoperative PSA ≥20	1.064	3.122	0.077	2.899
**Model constant**	-9.481	32.997	0.000	0.000

B, unstandardized regression weight; exp(B), odds ratio; GGG, Gleason Grading Group; MRI, magnetic resonance imaging; cT, clinical tumor stage; PPBC, percent of positive biopsy cores; PSA, prostate specific antigen.

The results of logistic regression, investigating predictors of pN1 status, are summarized in **Table 4**. Among the analyzed factors, three showed statistical significance: pT3-4 (OR = 5.315; *p* < 0.001), extracapsular extension (ECE) (OR = 4.795; *p* = 0.016), and LVI (OR = 23.905; *p* < 0.001). The analysis included 295 patients. Additionally, the results of linear regression, examining predictive factors for the number of positive lymph nodes in pN1 patients, are provided in **Supplementary Table 2** in the **Supplementary Material**. Due to the limited number of included patients (*n* = 74), the significance of these results is deemed unsatisfactory. However, for transparency and completeness, we have chosen to present these findings in the **Supplementary Material**.

**Table 4:** Logistic regression results with pN1 as a dependent variable.

Predictors	B	Wald	Statistical significance	Exp(B)
Postoperative GGG 3-5	-0.329	0.489	0.485	0.720
pT3-4	1.671	13.355	0.000	5.315
PPBC >50%	0.888	1.497	0.221	2.430
Preoperative PSA ≥20	0.071	0.018	0.893	1.074
ECE	1.568	5.769	0.016	4.795
PSM	0.434	0.767	0.381	1.544
PNI	-0.398	0.118	0.731	0.672
LVI	3.174	45.440	0.000	23.905
**Model constant**	-8.311	11.673	0.001	0.000

B, unstandardized regression weight; exp(B), odds ratio; GGG, Gleason Grading Group; pT, pathological tumor stage; PPBC, percent of positive biopsy cores; PSA, prostate specific antigen; ECE, extracapsular extension; PSM, positive surgical margin; PNI, perineural invasion; LVI, lymphovascular invasion.

Over a median follow-up period of 42 months, ranging from 3 to 102 months, 70 out of 423 patients (16.5%) experienced PSA failure, and 43 out of 414 patients (10.4%) exhibited disease progression. During this observation period, 10 out of 408 patients (2.5%) died, with 2 of these deaths attributed to PCa. The 5-year overall survival rate was 98.8%, and the 5-year cause-specific survival rate stood at 100%. Among 58 patients, the time from RP to BCR was known, with a mean duration of 19.2 months. In 42 patients classified as L0, the mean BCR-free survival period was 18.6 months, while in the remaining 16 patients classified as L1, it extended to 20.8 months with no statistically significant correlation observed.

## 4 Discussion

The objective of our research was two-fold. Primarily, we aimed to identify preoperative factors that are associated with the presence of LVI. This goal was driven by the aspiration to uncover preoperative markers that could aid in the identification of patients prone to LVI during postoperative examination. The rationale behind this endeavor is clear: if patients with evidence of LVI in their biopsy specimens can be identified preoperatively, it is imperative that they opt for RP or radiotherapy instead of embarking on active surveillance. In line with this, our findings point to several preoperative factors that demonstrate a significant correlation with LVI, providing valuable insights for clinical practice. As we consider future research directions, it is crucial to emphasize the ideal scenario: investigating the correlation of preoperative factors with LVI in biopsy specimens rather than relying solely on the final histopathological examination, as conducted in our study. This approach holds the potential to enhance risk stratification and further refine therapeutic decision-making in PCa management. The logistic regression identified preoperative PSA, PPBC, and GGG as robust predictors of LVI, consistent with findings from previous studies (5, 15, 25–32). Notably, we also identified MRI-based cT as a significant predictor of LVI, a factor not previously described in the literature to the best of our knowledge. While Kizilay et al. demonstrated a correlation between LVI and PIRADS score, Shin et al. (33) tumor visible of MRI was uncorrelated with LVI (p = 0.876) (33, 34). Our study suggests that cT, as determined by radiologists based on MRI, could be a valuable predictor for pL1 PCa. This factor may play a pivotal role in treatment planning, serving as an influential decision-making element.

Our secondary goal was to assess postoperative outcomes correlated with LVI, and among these outcomes, one of the most important discoveries was the relationship between LVI and the number of positive LNs in patients with nodal involvement. The mean number of positive LNs demonstrated a noteworthy discrepancy between patients without and with LVI in the pN+ group, registering means of 1.9 and 4.07, respectively, with a median of 1 and 3 (p < 0.001). This observation underscores the substantial clinical relevance of LVI within the context of PCa prognosis, particularly given the established association of nodal metastasis and poorer survival outcomes, as documented in prior studies (35–37).

Considering the pivotal finding of our study, which, to our best knowledge, is the first to establish a correlation between LVI and an elevated number of positive LNs in pN+ patients, it becomes increasingly apparent that the judicious selection of patients at higher risk of harboring LVI in their histopathological specimens is vital. Such patients could benefit from a more personalized therapeutic approach, potentially involving the consideration of LND as an integral component of their treatment strategy or/and a wider LND template. Notably, our study revealed instances of LVI in patients classified as low-risk according to their biopsy GGG, with 7.2% exhibiting LVI in the postoperative histopathological examination. These findings highlight the need for future investigations to focus on the analysis of preoperative factors that may predict LVI in biopsy specimens, as this represents the sole opportunity for risk stratification before surgery.

In essence, our study emphasizes the critical implications of LVI in PCa, underscoring the need for tailored approaches in patient management, particularly in selecting the appropriate candidates for radical treatment, even in cases traditionally considered low-risk. On the contrary, a study conducted by Semba et al. has brought to light a paradox where low-risk patients demonstrate favorable oncological outcomes despite the presence of LVI in final histopathological specimens (38). This observation underscores the pressing need for future research endeavors focused on unraveling the preoperative factors associated with LVI within a broader patient population. It is worth considering that future studies should place a particular emphasis on discerning the importance of LVI in intermediate-risk patients. This demographic represents a challenging ‘grey area’ where making treatment decisions can be notably complex. Determining whether to opt for radical treatment or active surveillance, whether to conduct lymph node dissection, and other critical decisions are all aspects that demand further exploration within this patient subgroup.

One of the inherent challenges in interpreting LVI lies in the difficulty of its proper assessment during histopathological examination. This intricacy may contribute to the substantial heterogeneity observed in the reporting of LVI across different studies, with incidences ranging widely from 3.6% to 53% (16, 17). The underlying reasons for such variability remain elusive and warrant further investigation. In our study, pL1 patients comprised 17.7% of the investigated population, aligning with the range described in the literature.

Our study is not without limitations. Notably, a majority of the patients in our cohort underwent open RP, which may not entirely align with current global trends favoring robotic or laparoscopic approaches. Additionally, the presence of missing data for some patients, albeit a consequence of the study’s large sample size, may have introduced a degree of selection bias. Furthermore, the retrospective and single-center nature of our study can impact its generalizability to broader clinical settings. It’s important to acknowledge that our study had limited power to conduct comprehensive survival analyses, as a substantial number of patients lacked complete data on survival and biochemical recurrence. Nevertheless, it’s essential to underscore that the primary aim of our investigation was not to extensively evaluate the effect of LVI on survival outcomes but rather to discern its preoperative and postoperative correlates. Furthermore, we did not conduct multivariate analysis specifically on postoperative outcomes associated with LVI. Consequently, the force of correlation between LVI and the number of positive LNs in pN+ patients was explored in univariate analysis only. Lastly, our study is rooted in clinical and histopathological data from PCa patients. However, it lacks specific data on the pathomorphological characteristics of the tumors. Therefore, a more comprehensive investigation of histological subtypes (such as intraductal adenocarcinoma or the cribriform pattern) could provide new insights into LVI across various subtypes, thus potentially improving the quality of the scientific evidence. Despite these limitations, it is essential to emphasize that postoperative outcome assessment, while in a univariate setting, contributes valuable insights to the broader understanding of LVI’s clinical implications in PCa.

In conclusion, our study adds a significant dimension to the understanding of LVI’s clinical implications in PCa. By revealing its association with the number of positive LNs, we emphasize the importance of identifying LVI as a prognostic factor, particularly in patients with lymph node involvement. Ideally, further studies should focus on the assessment of LVI during the biopsy. The preoperative factors linked to LVI provide actionable insights for risk stratification and clinical decision-making. Although the interpretation of LVI remains challenging and the reported incidences are highly variable, our study contributes to bridging the knowledge gap in this field and offers critical guidance for the diagnosis of PCa patients. Subsequent investigations should aim to unravel the underlying factors contributing to the observed heterogeneity in reporting LVI and to validate the practical utility of preoperative markers in clinical practice. Moreover, future research endeavors should focus on low- and intermediate-risk PCa patients. Emphasizing the assessment of LVI in biopsy specimens, rather than relying solely on RP specimens, would be crucial for enhancing preoperative risk stratification. Ideally, prospective, multi-center studies are warranted to provide a more comprehensive understanding of LVI’s clinical implications across diverse patient populations.

## 5 Conclusions

Our study highlights the pivotal role of LVI in PCa prognosis. Analyzing 861 PCa patients, we identified key preoperative predictors for LVI, including MRI-based cT, biopsy GGG, preoperative PSA, and PPBC. Notably, our research reveals a novel association between LVI and an increased number of positive LNs in pN+ patients in the univariate analysis. Despite study limitations, such as its retrospective nature and potential selection bias, our findings emphasize the significance of LVI in PCa, urging personalized approaches in patient prognosis-related decision-making. Future studies should delve into LVI’s implications for intermediate-risk patients and address the heterogeneity in LVI reporting across studies, as well as LVI as a possible biopsy finding.

## Data availability statement

The original contributions presented in the study are included in the article/**Supplementary Material**. Further inquiries can be directed to the corresponding authors.

## Ethics statement

The studies involving humans were approved by Ethics Committee of Wroclaw Medical University. The studies were conducted in accordance with the local legislation and institutional requirements. Written informed consent for participation was not required from the participants or the participants’ legal guardians/next of kin because the study has been conducted in accordance with the national legislation and institutional requirements.

## Author contributions

JK: Conceptualization, Methodology, Project administration, Writing – original draft. MŁ: Formal analysis, Methodology, Writing – original draft. MJ: Conceptualization, Writing – review & editing. AJ: Methodology, Writing – original draft. PS: Writing – review & editing, Formal analysis. AP: Writing – review & editing, Methodology. AL: Writing – review & editing. KK: Writing – review & editing. TS: Writing – review & editing, Conceptualization. AH: Investigation, Methodology, Writing – review & editing. BM: Funding acquisition, Supervision, Writing – review & editing.
