# Supplementary material for: The clinical meaning of lymphovascular invasion: preoperative predictors and postoperative implications in prostate cancer - a retrospective study
Source: Front Oncol. 2024 May 3;14:1349536. doi: 10.3389/fonc.2024.1349536 (PMC11099271; doi:10.3389/fonc.2024.1349536)
Supplement: Supplementary file 1 [file Table_1.docx]

Supplementary Material

The clinical meaning of lymphovascular invasion:
preoperative predictors and postoperative implications
in prostate cancer - a retrospective study

Jakub Karwacki^*^, Małgorzata Łątkowska, Michał Jarocki, Arkadiusz Jaworski, Przemysław Szuba, Adrian Poterek, Artur Lemiński, Krystian Kaczmarek, Agnieszka Hałoń, Tomasz Szydełko, Bartosz Małkiewicz^*^

*** Correspondence:** Bartosz Małkiewicz; bartosz.malkiewicz@umw.edu.pl; Jakub Karwacki; jkarwacki.md@gmail.com

# Supplementary Data

**SUPPLEMENTARY TABLE 1**. Tabular representation of the coding for individual variables included in respective logistic regression models. Model 3 showed the highest efficacy of predicting lymphovascular invasion (LVI).

| **Variable** | **Model 1** | **Model 2** | **Model 3** | **Model 4** | **Model 5** | **Model 6** |
| --- | --- | --- | --- | --- | --- | --- |
| MRI-based cT | cT1-2 = 0  cT3-4 = 1 | cT1-2 = 0  cT3-4 = 1 | cT1-2 = 0  cT3-4 = 1 | cT1-2 = 0  cT3-4 = 1 | cT1-2 = 0  cT3-4 = 1 | cT1-2 = 0  cT3-4 = 1 |
| cN | cN0 = 0  cN+ = 1 | cN0 = 0  cN+ = 1 | cN0 = 0  cN+ = 1 | cN0 = 0  cN+ = 1 | cN0 = 0  cN+ = 1 | cN0 = 0  cN+ = 1 |
| Biopsy GGG | 1-2 = 0  3-5 = 1 | 1-2 = 0  3-5 = 1 | 1-2 = 0  3-5 = 1 | 1-3 = 0  4-5 = 1 | 1-3 = 0  4-5 = 1 | 1-3 = 0  4-5 = 1 |
| Biopsy PSA | <15 = 0  ≥15 = 1 | <10 = 0  ≥10 = 1 | <20 = 0  ≥20 = 1 | <15 = 0  ≥15 = 1 | <10 = 0  ≥10 = 1 | <20 = 0  ≥20 = 1 |
| Preoperative PSA | <15 = 0  ≥15 = 1 | <10 = 0  ≥10 = 1 | <20 = 0  ≥20 = 1 | <15 = 0  ≥15 = 1 | <10 = 0  ≥10 = 1 | <20 = 0  ≥20 = 1 |
| PPBC | <50% = 0  ≥50% = 1 | <50% = 0  ≥50% = 1 | <50% = 0  ≥50% = 1 | <50% = 0  ≥50% = 1 | <50% = 0  ≥50% = 1 | <50% = 0  ≥50% = 1 |
| cT | cT1-2 = 0  cT3-4 = 1 | cT1-2 = 0  cT3-4 = 1 | cT1-2 = 0  cT3-4 = 1 | cT1-2 = 0  cT3-4 = 1 | cT1-2 = 0  cT3-4 = 1 | cT1-2 = 0  cT3-4 = 1 |

MRI, magnetic resonance imaging; cN, clinical lymph node status; GGG, Gleason Grading Group; PSA, prostate specific antigen; PPBC, percent of positive biopsy cores; cT, clinical tumor stage.

**SUPPLEMENTARY TABLE 2**. Linear regression results with the number of metastatic lymph nodes in pN1 patients as a dependent variable.

| **Predictors** | **B** | **SE** | **Beta** | **t** | **Statistical significance** |
| --- | --- | --- | --- | --- | --- |
| PPBC | 2.635 | 2.284 | 0.143 | 1.154 | 0.253 |
| Preoperative PSA | 0.064 | 0.022 | 0.337 | 2.864 | **0.006** |
| pT | -0.660 | 2.771 | -0.031 | -0.238 | 0.813 |
| Postoperative GGG | -0.036 | 0.559 | -0.007 | -0.064 | 0.949 |
| ECE | 1.564 | 2.283 | 0.090 | 0.685 | 0.496 |
| PSM | 0.406 | 1.539 | 0.032 | 0.264 | 0.793 |
| PNI | 0.307 | 5.571 | 0.007 | 0.055 | 0.956 |
| LVI | 1.707 | 1.607 | 0.127 | 1.063 | 0.292 |
| **Model constant** | -2.919 | 13.278 | - | -0.220 | 0.827 |

B, unstandardized regression weight; SE, standard error; PPBC, percent of positive biopsy cores; PSA, prostate specific antigen; pT, pathologic tumor stage; GGG, Gleason Grading Group; ECE, extracapsular extension; PSM, positive surgical margin; PNI, perineural invasion; LVI, lymphovascular invasion.
